# Antimicrobial practices among small animal veterinarians in Greece: a survey

**DOI:** 10.1186/s42522-020-00013-8

**Published:** 2020-05-18

**Authors:** George Valiakos, Eleni Pavlidou, Christos Zafeiridis, Constantina N. Tsokana, Victor J. Del Rio Vilas

**Affiliations:** 1 Faculty of Veterinary Science, University of Thessaly, Karditsa, Greece; 2 Centre for Universal Health, Chatham House, London, UK; 3 Asclepius One Health Platform, Athens, Greece; 4 Hellenic Republic Ministry of Rural Development & Food, Athens, Greece

**Keywords:** Antibiotics usage, Antimicrobial resistance, Greece, Small animal veterinarians, Survey

## Abstract

**Background:**

The inappropriate use of antibiotics is a major issue in clinical practice in Greece with serious implications for public health and animal health. The purpose of the present study was to provide a first insight into the use of antibiotics by small animal practitioners in Greece and assess their compliance with general rules for the rational use of antibiotics. This is the first survey of its kind in Greece.

**Methods:**

A questionnaire was designed to collect basic information on the use of antibiotics by pet veterinarians. The questionnaire was sent to a total of 70 veterinarians mainly operating in the region of Attica, a region that comprises almost 50% of the Greek population and where veterinarians are engaged solely in small animal practice. The questionnaire consisted of 37 closed questions dealing with various aspects on the use of antibiotics.

**Results:**

The majority of practitioners report cases where the pet owner initiated antibiotic treatment without veterinary prescription. Almost every clinician reported owner-compliance challenges. Regarding microbiological analysis, 73% of respondents initiate empirical treatment while waiting for laboratory results or use antibiogram only when the treatment is unsuccessful. Eighty-eight per cent declared to use antimicrobials postoperatively in clean surgical procedures. Different types of antimicrobials and treatment durations than the ones proposed by guidelines on rational use of antibiotics are preferred for various organ systems e.g. in urinary and gastrointestinal infections.

**Conclusions:**

Our findings suggest the need for guidelines on antibiotic use in small animal practice in Greece, and the deployment of systematic surveillance on antimicrobials use and resistance to inform the initial choice of antibiotics upon local antimicrobial resistance profiles. Targeting the other end of the problem, pet owners, our findings indicate the need to educate them on the rational use of antibiotics and, critically, stop antibiotic availability without prescription.

## Background

The use of antibiotics is essential in the treatment of infectious diseases in pets. However, the day-to-day use of antibiotics has led to the emergence of significant bacterial populations resistant to many classes of antibiotics, raising important public health issues [1, 2]. Antibiotic-resistant bacteria not only make it difficult to apply effective treatment to the patient, but can themselves 1) be transmitted to other humans and animals, and 2) transmit their antibiotic resistant properties to other types of bacteria through horizontal genetic transfer, further complicating the treatment of other diseases. These phenomena occur in the clinical practice of pets for a number of important pathogenic microorganisms such as *Staphylococcus* spp., *Campylobacter* spp., *Salmonella* spp. and *Escherichia coli* [3]. The amount of antibiotics used in pet animals in Europe is small in comparison to that used in livestock [4], however the close contact of pets with their owners facilitates the transmission of multidrug-resistant organisms between humans and their pets [5–11].

The spread of antimicrobial resistance (AMR) has led to the rationalization of antibiotic use in humans and animals. To help achieving this, information on the use practices of antibiotics by clinicians and on locally developed antibiotic resistance patterns is needed. This evidence can help identify problems arising from wrong practices and inform appropriate recommendations and incentives to address them. At the same time, therapeutic protocols can be more stringently enforced against local circulating bacteria, using specific narrow-spectrum antibiotics instead of last generation antibiotics of broad-spectrum that are more likely to promote AMR in a broader group of bacteria [12].

The inappropriate use of antibiotics is a major issue in human clinical practice in Greece with serious implications for public health [13–15]. Greece crowns the list of EU countries regarding human antibiotic consumption per capita and reports 40% of human infections due to antibiotic-resistant bacteria compared to EU’s average of 20% [16]. Investments in public health actions to tackle AMR are still insufficient [16]. To date, only a minority of EU/EEA countries have implemented national action plans against AMR and defined a monitoring and evaluation process. In Greece, a national action plan is being prepared (www.moh.gov.gr, in Greek). However, use of antibiotics in veterinary medicine, and especially in small animal practice, is not yet being formally considered as part of the Greek public health efforts to address AMR.

The purpose of the present study was to provide a first insight into the use of antibiotics by small animal practitioners in Greece, and a preliminary assessment of the trends of Greek pet veterinarians regarding the rational use of antibiotics in small animal practice as proposed in relevant guidelines contacted in other countries but not in Greece [17]. The information collected aims to inform current efforts towards the development and implementation of Greece’s national plan on AMR. To the best of our knowledge, this is the first survey of its kind in Greece.

## Materials and methods

A questionnaire was designed to collect information on the use of antibiotics by veterinarians. The questionnaire was designed to be simple, with few questions so as not to discourage participation. The questionnaire was sent to a total of 70 veterinarians, a convenience sample built from an initial list of pet veterinarians attending the 1st Hellenic One Health forum in Athens in 2018 (*n* = 30) and extended through a snowball sampling approach, using data provided by the Hellenic Veterinary Association (HVA). The sampling targeted veterinarians mainly operating in the region of Attica (*n* = 50, 71% of them), a region that comprises almost 50% of the Greek population and where veterinarians are engaged solely in small animal practice, which is the main focus of this study. Small animal veterinarians are approximately 400 in the Attica region (Small Animal Veterinarians of Attica Association, www.skmza.gr), considered to be approximately 40% of the pet veterinarians in the country (source: HVA). The rest (*n* = 20, 29%) of the veterinarians were selected from other areas of mainland Greece, engaged also solely in small animal practice.

An email was sent to the veterinarians with a cover letter explaining the purpose of the study and including a link to an online questionnaire. The questionnaire could be completed anonymously on a voluntary basis over a period of 2 months.

The questionnaire was written by members of the research team and is consistent with questionnaires used in similar studies in other countries, to allow comparison of the results [18–20]. It consists of 37 closed questions. Most of the questions aim to identify the frequency of at which an events of interest, e.g. proportion of antibiotic treatment non-compliance by pet owners (never 0%, sometimes 30%, frequently 70%, always 100%). The first part of the questionnaire (18 questions) gathers personal information about the clinician, antibiotic use in clinical practice, and their postoperative use in surgical procedures. The second part (11 questions) includes questions on the use of different types of antibiotics by group of diseases and organ system; and the third part (8 questions) enquires about the duration of therapeutic protocols. Frequency tables and the Fisher's exact test, to assess the association between years of experience and various aspects of the clinical practice and use of antibiotics, were run in SPSS V.18.0.

## Results

### General demographic data

Forty-eight (68.6%) veterinarians responded to the questionnaire; most of them (*n* = 38, 79% of respondents) come from the Attica region. Tables 1, 2 and 3 show the answers to the three categories of questions. The majority of respondents taking part in the survey (*n* = 31, 65%) were working in pet clinics with no more than 2 staff veterinarians. At total of 37 (77%) were male. Almost half (*n* = 21, 44%) were 36–50 years old, two were 25–35 years old and the remaining (*n* = 25, 52%) were more than 50 years of age. A total of 37 of the respondents (77%) had more than 15 years of professional experience.

**Table 1:** Questionnaire responses (frequency and percentage) by the 48 respondents

Question	Options	Respondents	
		Frequency	(%)
Gender	Male	37	77.08
	Female	11	22.92
Age	25–35	2	4.17
	36–50	21	43.75
	> 50	25	52.08
Years of experience	1–15	11	22.92
	16–25	26	54.16
	> 26	11	22.92
Number of Vets	1	16	33.33
	2	15	31.25
	> 2	17	35.42
Written antibiotic policy	Yes	22	45.83
	No	26	54.17
How often do you encounter owner-initiated treatments before a case is presented to the practice?	Never (0%)	5	10.42
	Sometimes (30%)	31	64.58
	Frequently (70%)	12	25.00
How often do you prescribe combinations of different antimicrobials?	Infrequently	32	66.67
	Frequently	16	33.33
How often do you encounter owner compliance challenges?	Frequently (70%)	46	95.83
	Always (100%)	2	4.17
Choice of the antibiotic is based mainly on	Bibliography (e.g. therapy handbooks)	35	72.92
	Leaflet indications	4	8.33
	My own professional experience	9	18.75
How often do you use microbiological analysis and antimicrobial susceptibility testing in cases where use of antibiotics is needed?	Sometimes (30%)	27	56.25
	Frequently (70%)	20	41.67
	Always (100%)	1	2.08
While you wait for the laboratory results, how often do you use antibiotics?	Never (0%)	3	6.25
	Sometimes (30%)	16	33.33
	Frequently (70%)	17	35.42
	Always (100%)	12	25.00
What is your preferred method of selecting your antimicrobial, in relevance to antibiogram results?	Empirical, whilst awaiting antibiogram	24	50.00
	Empirical first, antibiogram if unsuccessful	9	18.75
	Antibiogram first	13	27.08
	I rarely use antibiogram	2	4.17
Do you keep a client’s record for antibiotics prescribing?	Yes	15	31.25
	No	33	68.75
If no, please state the reason	Lack of Time	31	64.58
	Not important	1	2.08
	Both of the above	12	25.00
	Other reasons	4	8.34
How often do you weight the animal before prescribing antibiotics?	Frequently (70%)	5	10.42
	Always (100%)	43	89.58
How often do animals return because of antibiotic treatment failure?	Never	8	16.67
	Sometimes (1–3 times a year)	33	68.75
	Frequently (more than 3 times a year)	7	14.58
How often do you use postoperative antibiotics in clean surgical operations?	0% of cases	6	12.50
	1–10% of cases	13	27.08
	11–50% of cases	5	10.42
	51–90% of cases	6	12.50
	> 90% of cases	18	37.50
For which reason do you often (more than 50% in previous question) apply postoperative antibiosis for this type of operation?	Just the typical procedure	27	56.25
	Operations last more than 90 min	2	4.17
	Frequent issues with aseptic procedures	7	14.58
	Frequent postoperative infections	3	6.25
	Other reason	9	18.75

**Table 2:** Percentage of respondents’ type of antimicrobial usage for different organs systems. The cells in bold represent the first-choice antimicrobial for every organ system

	Skin	Ear	Urinary	GIT	Reproductive	Respiratory	Sepsis	Eye
Penicillins	25,53	11,53	8,89	8,12	**25,00**	3,77		**25,00**
Cefalosporins 1st,2nd gen	**31,91**	7,69	11,11	5,41	12,50	7,55	16,67	18,75
Cefalosporins 3rd,4th gen	10,64	15,38	11,11	2,70	6,25	11,32	22,22	18,75
Aminoglycosides	2,13	**34,62**	6,67	2,70	6,25	1,89	11,11	
Quinolones	4,26	15,38	**46,67**	5,41	6,25	7,55	**33.33**	6,25
Tetracyclines		3,85		2,70	12,50	**32,07**		12,50
Sulphonamides			13,33	**32,43**	**25,00**	7,55		
Lincosamides	12,77		2,22	2,70		7,55	5,56	
Macrolides	6,38	3,85		2,70	6,25	16,98	11.11	6,25
Nitroimidazoles		3,85		**32,43**				
Other	6,38	3,85		2,70		3,77		12,50

**Table 3:** Duration of treatment (columns) for different organ systems (rows). Cells show the frequency and percentage of responses (in brackets). The cells in bold represent the most used duration of treatment for every organ system

Organ system	Treatment length				
	Less than 3 days	3–7 days	8–14 days	15–21 days	Over 21 days
Skin	1 (2.08)	2 (4.17)	16 (33.33)	**18 (37.50)**	11 (22.92)
Ear	0 (0.00)	3 (6.25)	**27 (56.25)**	12 (25.00)	6 (12.50)
Urinary	0 (0.00)	4 (8.33)	**23 (47.92)**	18 (37.50)	3 (6.25)
GIT	2 (4.17)	**23 (47.91)**	18 (37.50)	3 (6.25)	2 (4.17)
Reproductive	1 (2.08)	12 (25.00)	**30 (62.50)**	4 (8.33)	1 (2.08)
Respiratory	0 (0.00)	8 (16.67)	**29 (60.41)**	11 (22.92)	0 (0.00)
Sepsis	0 (0.00)	1 (2.08)	14 (29.17)	14 (29.17)	**19 (39.58)**
Eye	1 (2.08)	**28 (58.33)**	13 (27.08)	6 (12.50)	0 (0.00)

### Use of antibiotics

Almost half of the veterinarians (*n* = 22, 46%) answered that they have a written antibiotic policy in their practice. Forty-three respondents (90%) answered that pet-owners already administered antibiotics before they bring the animal to the practice (65% “sometimes”, 25% “frequently”), and every clinician reported treatment compliance challenges. Regarding microbiological analysis and antimicrobial susceptibility testing, 20 respondents (42%) use them “frequently” and 27 (56%) “sometimes”. Only 6 % of the clinicians who make use of such laboratory support declared to ‘always’ wait for the results before prescribing an antibiotic. In a similar question, focusing on what they “prefer” to do (even though they may be forced to do differently in many cases) 13 participants (27%) responded that they prefer not to use antibiotics while awaiting the laboratory results. The remaining 35 participants (73%) prefer to adopt an empirical treatment (i.e. a clinical “educated guess” in the absence of accurate diagnosis) while waiting for the laboratory results or use antibiogram only when the empirical treatment is unsuccessful. Forty-two (88%) clinicians declared to use antimicrobials postoperatively in clean surgical procedures, half of them in more than 50% of the cases. Most of them answered that “this is the typical procedure”.

Based on the collected data, the veterinarians do not frequently prescribe combinations of different antibiotics (67% infrequently, 33% frequently). In order to understand what antibiotics are mainly used to treat infections involving specific organ systems, veterinarians were asked to indicate the class of antimicrobials or the active compound of preference. The results showed that for urinary tract infections veterinarians prescribe mainly (fluoro) quinolones, for cutaneous diseases cephalosporins, for respiratory diseases tetracyclines, and for gastrointestinal diseases sulphonamides and nitroimidazole (Table 2). Quinolones are the preferred type of antimicrobial used in cases of sepsis, penicillins in eye infections, and aminoglycosides in ear infections. Finally, for reproductive diseases, sulphonamides and penicillins are mainly used.

### Treatment periods

Treatment periods were variable depending on the organ system (Table 3). Skin infections were treated for more than 15 days by most of the clinicians (60%). The majority of the clinicians treat urinary, reproductive, ear, and respiratory diseases for more than 8 days (92, 73, 94, 83% respectively). Gastrointestinal Tract (GIT) and eye infections are treated mainly for more than 3 days; 48% of respondents treat GIT infections for more than 8 days. Finally, cases of sepsis are treated usually for more than 15 days.

### Statistical analysis

Fischer's exact tests did not return any significant association between years of experience (less than or greater than 15 years) and having written antibiotic policy, the habit of weighing animals before prescribing antibiotics, preferred method of selecting antibiotics, and postoperative use of antibiotics in clean surgical operations (Table 4).

**Table 4:** Correlation between veterinarians’ years of experience and aspects of small animal practise

		Years of experience		
		1–15	> 15	
Written antibiotic policy	Yes	3	19	Fisher Exact *p* = 0.1887
	No	8	18	
Weighing animals before prescribing antibiotics	Always (100%)	8	35	Fisher Exact *p* = 0.0716
	Often (100%)	3	2	
Preferred method of selecting antibiotics	Empirical, whilst awaiting antibiogram	6	18	Fisher Exact *p* = 0.6657
	Empirical first, antibiogram if unsuccessful	1	8	
	Antibiogram first	4	9	
	I rarely use antibiogram		2	
Postoperative use of antibiotics in clean surgical operations	0% of cases		6	Fisher Exact *p* = 0.5094
	1–10% of cases	2	11	
	11–50% of cases	1	4	
	51–90% of cases	2	4	
	> 90% of cases	6	12	

## Discussion

The 38 veterinarians from Attica who completed the questionnaire represent approximately 10% of the population of small animal practitioners in the area of Attica (source: HVA). The 48 veterinarians represent approximately 5% of the small animal practitioners in Greece. This sample size is similar to that of other early studies conducted elsewhere (10% in Italy, 14% in UK, 16% in South Africa) [18–20]. This sample size corresponds to a 14–15% margin of error for the region of Attica as well as Greece, an acceptable number for a first preliminary survey. In order to draw safer conclusions for the entire Greek territory, the study must be expanded to a significant number of veterinarians in other areas producing a more representative set of results for the whole country. Participation was encouraging with a response rate of 69%.

Almost half of the veterinarians answered that they have a written antibiotic policy in their practice. This compares to 27% in South Africa and 30% in Denmark [20, 21]. In the UK, 97% of practicing veterinarians responded in earlier studies that they did not use fixed protocols [19]. Having written antibiotic protocols is important as it promotes optimal treatment, and helps limit the prescription of antimicrobials of critical clinical importance in human medicine, e.g. fluoroquinolones and cephalosporins of 3rd and 4th generation which should only be used following culture and susceptibility testing. Despite the apparent large proportion of respondents affirming that they have written protocols, it is important to state that there are no guidelines on the use of antibiotics by any veterinary authority in Greece. Practitioners developtheir own protocols, possibly leading to heterogeneity in the antibiotic treatment of clinical cases, and over-prescription of “last resort” antimicrobials when the practitioner is not up to date in the rational use of antibiotics. This likely heterogeneity is compounded by the lack of geographically explicit AMR surveillance data to inform practitioners’ protocols.

Of grave concern is that the majority of pet-owners administer antibiotics before they bring their animals to the practice. Pet owners are probably using antimicrobials without taking into consideration their spectrum of activity or resistance profiles, and they are either using remnants from previous visits to a veterinarian or even antibiotics used in human medicine. In Greece, there is legislation stipulating that antibiotics should only be prescribed by health professionals. Eurobarometer data on human antibiotic use for Greece in 2013 and 2016 showed that 16 and 20%, respectively, of all users do so without a medical prescription, the highest percentages in the EU [22]. This figure decreased to 9% in 2018, indicating success in the implementation of policy recommendations as suggested by the European Commission. However, Eurobarometer data indicates that Greece is still one of the EU countries with the lowest awareness and understanding of antibiotic use by the general population [23].

Use of antimicrobial combinations is not frequent; our results are similar to those of a study in Italy [18], but different from a study in the UK that showed that antibiotic combinations were commonly prescribed [18]. We did not enquire further about the specific combinations; these should be investigated in a next extended survey. Antimicrobial combination is suggested to achieve synergistic or additive results, allowing lower doses of compounds and thus preventing emergence of antimicrobial resistance [24].

Almost every clinician reported owner-compliance challenges; this usually has to do with pet owners stopping treatment when clinical signs disappear (a general rule of thumb is to continue treatment for 1–2 days beyond resolution of clinical signs and for 2 weeks in serious deep pyoderma problems) [17]. This should be further investigated in a future more extensive study, as it is important to educate pet owners accordingly.

Most of the veterinarians answered that they use microbiological analysis and antimicrobial susceptibility testing, alas with variable frequency; most (56%) answered “sometimes” and 41% answered “frequently”. A similar result was obtained by Barbarossa et al. in Italy [18] (69 and 20%, respectively, while 2% answered “always”). However, only 27% of our respondents answered that they prefer to wait for the antibiogram results before using any antibiotics, and 94% answered that they have to use antimicrobials while waiting for the laboratory results, with variable frequency (from “sometimes” to “always”). These answers indicate that the first choice of antibiotic is usually made empirically; most answered that they based their choice on bibliography (e.g. therapy handbooks), which we assume is their main source for compiling their written antibiotic policies. The empirical use of antimicrobials should be avoided, with the exception of infections causing pain or discomfort or for complicated or life-threatening infections [25]. There is an urgent need to reinforce the use of laboratory analysis in cases where multiresistant bacteria may be implicated or there is a risk of increasing antimicrobial resistance, e.g. *Staphylococcus* spp. and *E. coli* in cutaneous and urinary tract infections, respectively. For those cases where empirical use of antimicrobials cannot be avoided, up-to-date information on local resistance profiles would be critical to narrow down antibiotic choice.

Most veterinarians (85%) do not see animals returning to the practice because of antibiotic treatment failure. This may explain why 69% of respondents answered that they do not keep their clients’ record of antibiotics prescription, believing it is not important or due to lack of time. Although most respondents weigh the animals before prescribing antibiotics, answers regarding the types of antibiotics used and duration of treatment per organ system are concerning.

Forty seven percent of the veterinarians treat urinary tract infections with (fluoro)quinolones. Relevant guidelines suggest the use of (fluoro) quinolones only in upper urinary tract infection (pyelonephritis) in dogs and cats; most cases of cystitis should be treated with amoxicillin or trimethoprim/sulphonamides, optimally after laboratory results confirm the presence of bacteria [17]. The frequent isolation of ESBL-producing *E. coli* from the urinary tract of dogs and cats is of increased significance due to the risk of infecting owners and veterinarians. Thus, it is important to limit their advance with increased microbiological monitoring and rational antibiotic use.

In the present survey, respondents answered that cephalosporins of 1st and 2nd generation, and penicillins are the preferred choices for dealing with skin infections, which is encouraging. Cutaneous infections are important in terms of antimicrobial resistance, with methicillin resistant strains of *Staphylococcus aureus* and *Staphylococcus pseudintermedius* presenting a serious threat to animal health due to the increased risk of treatment failure. These bacteria are recognised nosocomial pathogens and can spread between animal patients via contamination of the hospital environment, invasive procedures and veterinary personnel [26]. As for ESBL-producing *E. coli*, it is important to limit the genetic pressure towards antimicrobial resistance development through microbiological monitoring of all cutaneous infections and rational use of antibiotics.

For respiratory infections, tetracyclines are the preferred choice, which is consistent with guidelines, with macrolides being second choice, where penicillins should be. In reproductive infections, penicillins and trimethoprim/sulphonamides are preferred by half of the respondents, as recommended by the guidelines. A concerning finding is that, in GIT infections, metronidazole is used by 1/3 of the respondents as the first choice antimicrobial; metronidazole is critical in managing *Clostridium difficile* infections in humans, and countries like Denmark have managed to reduce its use in veterinary medicine to nearly zero levels after a campaign initiated in 2012 [17]. For the rest of the system organs (eye, ear, sepsis, reproduction) first choice antibiotics are near the recommended ones.

Different treatment durations than the ones proposed by guidelines on rational use of antibiotics were reported for various organ systems. Almost every veterinarian (91,67%) reported treatment durations of more than 8 days for urinary tract infections, while the recommended period is for 3–5 days [17]. For respiratory diseases, more than 83% of the practitioners stated a usual treatment duration of more than 8 days (vs. the recommended 5–7 days duration). For GIT infections, almost half of the respondents (48%) apply antimicrobials for 8–14 days, while the general recommendations are for 3–7 days [14]. For the other organ systems (skin, ear, reproduction, sepsis and eye) reported treatment periods were similar to those recommended by the guidelines. 

Regarding postoperative use of antibiotics in clean surgical procedures, half of the veterinarians reported their use in more than 50% of their cases, answering that they consider this to be “the typical procedure”. It is generally accepted that low-risk patients (healthy or with localised disease), and apyrexic patients with serious systemic illness, undergoing a clean or clean-contaminated procedure do not require antibiotic prophylaxis [27]. Hence, it is a subject in which most of the respondents appear unaware of best practices. We must of course take into account that pet owners in Greece are usually surprised to learn that their pet will not receive antibiotics prior to routine surgery, and that they will be sent home without oral antibiotics.

## Conclusions

Our findings suggest a number of recommendations towards the rationalization of antibiotics use in small animal practice in Greece.

Firstly, practitioners need guidance on the subject. Official guidelines have been implemented by some countries, e.g. Denmark, with encouraging results showing overall decrease of the use of antimicrobials and especially those of importance for human health such as cephalosporines of 3rd and 4th generation and metronidazole [21]. Guidelines for the rational use of antibiotics in small animal practice should be compiled by the country’s relevant authorities, e.g. the professional Veterinary Association, or the relevant government department. Moreover, these guidelines should be included in the veterinary undergraduate curriculum. These guidelines should have a “per organ system” recommendations approach, so as to highlight the most important needs for changes in small animal practice; regular data collection (using appropriate questionnaires) by the veterinarians should be used to detect periodically large discrepancies, and adjust the guidelines accordingly.

Secondly, there should be systematic antimicrobial use and AMR surveillance to inform the initial choice of antibiotic upon local resistance profiles. As sensitivity testing in general practice tends to be restricted to the most difficult cases there is an overestimation of resistance levels and their severity. Surveillance should comprise a larger number of uncomplicated or first-time infections leading to improved monitoring quality and giving a more accurate picture of resistance development generally. This data should regularly update the previously compiled guidelines, redefining empirical therapy protocols and ensuring optimized treatments using the most appropriate, locally relevant, narrow-spectrum antibiotics per organ system infections.

Finally, it is important to reduce the availability of antibiotics without prescription to pet owners and educate them about antimicrobial resistance and the importance of rational use of antibiotics on their pets. In a truly One Health approach, the veterinarian should be able to explain to the animal owner in simple terms why an antimicrobial is given, the importance of completing treatment, how antimicrobial resistance develops and its significant impact on the pet’s health and, critically, on public health. Similarly, public health campaigns should widen their message to include the AMR risks from the indiscriminate use of antibiotics in animals.

## Data Availability

The datasets used and/or analysed during the current study are available from the corresponding author on reasonable request.

## Abbreviations

AMR: Antimicrobial resistance
GIT: Gastrointestinal tract
HVA: Hellenic Veterinary Association
